# Tools to evaluate medication management for caregivers of people living with dementia: A systematic review

**DOI:** 10.1111/hex.13318

**Published:** 2021-07-21

**Authors:** Melissa Gench, Mouna J. Sawan, Aili Langford, Danijela Gnjidic

**Affiliations:** ^1^ School of Pharmacy, Faculty of Medicine and Health The University of Sydney Sydney New South Wales Australia; ^2^ Charles Perkins Centre The University of Sydney Sydney New South Wales Australia

**Keywords:** caregiver, dementia, medication management, transitions of care

## Abstract

**Background:**

Caregivers often undertake medication management for people living with dementia without formal training. There is a need to evaluate caregiver medication management practices for people living with dementia to identify and address the key issues that contribute to caregiver burden.

**Objectives:**

This study aimed to identify and summarize approaches that evaluate medication management for caregivers of people living with dementia and appraise caregiver's involvement in aspects of medication management.

**Search Strategy:**

A systematic search was undertaken in five databases: Medline, Embase, PsycINFO, Scopus and International Pharmaceutical Abstracts. Studies written in English that contained tools and surveys that evaluated aspects of medication management for caregivers of PWD were included.

**Results:**

A total of 10 studies were included. Medication selection was assessed in six studies, supply and monitoring/review was captured in seven studies, with administration assessed in nine studies. Caregivers were commonly involved in decision‐making for medication changes (77.1%–86.8%) and in the ordering (55.9%–86.0%) and collection (87.0%–92.4%) of medications. Reported caregiver involvement in medication administration showed a wide range (44%–94.7%) between the studies. Challenges in administration were commonly related to polypharmacy and dosage regimen complexity.

**Conclusions:**

Current tools capture specific aspects of medication management, with medication administration the most evaluated aspect of medication management. Future research is needed to develop a tool to holistically evaluate the complexities of medication management for caregivers of people living with dementia to minimize adverse events at transitions of care.

**Public Contribution:**

From the authors' previous research, caregivers highlighted the need to address key issues in medication management for people living with dementia.

## 1 INTRODUCTION

People living with dementia are commonly exposed to polypharmacy and potentially inappropriate medication use, which have been associated with increased medication burden, cognitive and functional decline and hospitalization.^1^, ^2^ The caregiver plays an important role in overseeing management of medications, which increases as the cognitive function of the individual with dementia declines.^3^, ^4^ Approximately 54% of caregivers are involved in medication management for the person living with dementia, which increases to 90% of caregivers in the later stages of dementia.^3^, ^5^

Medication management for individuals living with dementia is a challenging aspect of caregiving.^6^ Caregivers may conduct approximately 10 medication management activities daily,^7^, ^8^ which contributes significantly to caregiving burden.^6^ Medication management involves caregivers selecting medications for the person living with dementia, supplying medications by obtaining new supplies of medications, preparing and administering medications and monitoring for adverse effects for the person living with dementia.^9^, ^10^ The complexities of managing medications increase with the progression of their care recipient's dementia diagnosis^11^ and at transitions of care, with communication failures and delayed, poorly timed discharges contributing to preventable medication‐related problems.^12^, ^13^, ^14^ Medication changes are not always communicated to people living with dementia, their caregivers or their primary healthcare providers.^15^ This can lead to inappropriate medication management, which is particularly common during transitions of care.^15^ Tailored medication management guidance provided to caregivers of persons with dementia is important to reduce the risks of medication‐related problems and adverse events, including hospitalization.^6^, ^10^

A priority of the World Health Organization in their global patient safety challenge ‘Medication Without Harm’ is vulnerable patients, which includes people living with dementia.^16^ Given that medication management is a key role for caregivers of people with dementia, it is important to evaluate the medication management aspects that caregivers are involved in, to identify the support and education that caregivers need to ensure that medications are managed safely.^17^ At present, there is limited research on available tools and surveys that measure caregiver medication management for people living with dementia. The aim of this systematic review is to identify and summarize approaches that evaluate medication management in caregivers of people living with dementia and appraise caregivers' involvement in aspects of medication management.

## 2 METHODS

This systematic review was conducted in accordance with the Preferred Reporting Items for Systematic Reviews and Meta‐Analyses (PRISMA).^18^ The review has not been registered, nor was a protocol prepared, as per PRISMA guidelines.

### 2.1 Search strategy

A systematic search of five electronic databases for relevant articles was undertaken on Medline, Embase, PsycINFO, Scopus and International Pharmaceutical Abstracts from inception to April 2020. Identification of approaches used to evaluate medication management for caregivers of people living with dementia was the primary outcome. The following search terms, and their variations, were split into three themes for database searching: (dementia* OR alzheimer* OR frontotemporal lobar degeneration or lewy body) AND (caregiv* or care giver* or carer* or caretaker* or family involvement) AND ([medicat* OR drug* OR prescription* OR medical*] adj4 [manag* OR treat* OR administ* OR adher* OR monitor* OR knowledge* OR storage OR appointment* OR therap* OR use*]). The same keywords were used throughout all database searches, with minor variations using the relevant database thesaurus—MeSH or Emtree terms. Following deduplication, articles were screened for relevance by title and abstract by two reviewers (Melissa Gench and Aili Langford). The remaining full‐text articles were assessed for eligibility independently by two reviewers (Melissa Gench and Aili Langford). Where there was disagreement between reviewers, discrepancies were resolved by a third reviewer (Melissa Gench).

### 2.2 Eligibility criteria

Studies were included if they were (1) original studies of quantitative design, (2) studies of tools and/or surveys identifying any aspect of medication management for caregivers of people living with dementia, (3) informal caregivers of people living with dementia, (4) conducted in any setting, (5) written in English and (6) in any time frame. For the purpose of this review, caregivers were defined as informal providers of care, such as familial relatives or friends, who are primarily responsible for caring for the person with dementia, and of which medication management comprises an aspect of caregiving.^19^ Medication management was defined as the ways in which caregivers select, supply, administer and monitor (including review) medications to provide optimal health outcomes for the person with dementia.^9^, ^10^ Studies were excluded if caregivers were not of people living with dementia, were formal, paid caregivers (e.g., nurses) or if tools and surveys did not evaluate caregiver medication management.

### 2.3 Data extraction and analysis

Data extraction was undertaken by Melissa Gench using a standardized extraction tool. Data extracted included (1) author, year and country, (2) study design; (3) number of caregiver participants; (4) mean age; (5) tool/survey name, description and focus of medication management; and (6) key findings in relation to caregiver medication management. A content analysis of the tools and surveys within the included studies was undertaken to extract reliability and validity information.^20^

## 3 RESULTS

### 3.1 Search results and study characteristics

**Figure 1:**
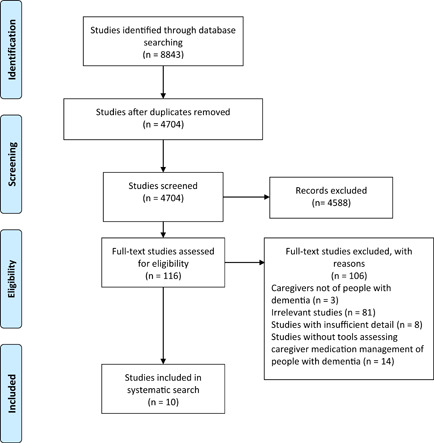
Preferred Reporting Items for Systematic Reviews and Meta‐Analyses (PRISMA) flowchart

In total, 8843 studies were identified through the database search. After duplicate removal, 4704 studies were screened for eligibility based on article title and abstracts. The process resulted in 116 studies for full‐text screening. Inclusion and exclusion criteria were applied, which led to 10 studies for inclusion in this review (see Figure 1). All studies were conducted in the United States of America^5^, ^21^, ^22^, ^23^, ^24^, ^25^, ^26^, ^27^, ^28^, ^29^; however, one study was a multicountry study.^22^ All studies were conducted in a community setting,^5^, ^21^, ^22^, ^23^, ^24^, ^25^, ^26^, ^27^, ^28^, ^29^ with the addition of one study that was also conducted in a long‐term care setting.^22^ Study designs included cross‐sectional studies (*n* = 8), unblinded randomized‐controlled trials (*n* = 1) and mixed‐methods studies (*n* = 1), with sample size ranging from 53 to 1369 participants (Table 1). Only 1^22^ out of the 10 included studies reported the stage of dementia diagnosis for their participants.^5^, ^21^, ^22^, ^23^, ^24^, ^25^, ^26^, ^27^, ^28^, ^29^ Medication selection was an aspect of medication management reported in 6 out of 10 studies,^21^, ^23^, ^26^, ^27^, ^28^, ^29^ supply^5^, ^21^, ^23^, ^24^, ^26^, ^27^, ^28^ and monitoring/review^5^, ^22^, ^23^, ^25^, ^26^, ^27^, ^29^ was captured in seven studies and administration was reported in nine studies.^5^, ^21^, ^22^, ^23^, ^24^, ^25^, ^26^, ^27^, ^28^ A total of seven tools capturing one or more aspects of medication management were identified from these studies: Caregiver Confidence in Sign/Symptom Management scale, Family Caregiver Medication Administration Hassles Scale (Hassles scale), Medication Complexity Index, Medication Deficiency Checklist, Medication Management Instrument for Deficiencies in the Elderly, Medication Risk Questionnaire and the Medication‐Saving Behaviors scale.^5^, ^21^, ^22^, ^23^, ^24^, ^25^, ^26^, ^27^, ^28^, ^29^ Some tools—the Hassles scale, the Caregiver Confidence in Sign/Symptom Management scale and the Medication Risk Questionnaire—were used across multiple studies.^21^, ^23^, ^25^, ^26^, ^27^, ^29^ The Hassles scale was used to measure caregiver burden in all aspects of medication management.^21^ The Caregiver Confidence in Sign/Symptom Management scale appraised caregiver confidence in selecting, administering, monitoring and reviewing medications for people living with dementia.^25^ The Medication Risk Questionnaire identified challenges in medication administration, supply and review.^23^

**Table 1:** Characteristics of studies evaluating caregiver medication management for people living with dementia (*n* = 10)

Author, year, country	Study design	Total number of caregiver participants	Mean age (years)	Tool/survey name, description and focus for medication management	Key findings in relation to caregiver medication management
George and Steffen, 2017, USA^21^	Cross‐sectional	53	53.4	Family Caregiver Medication Administration Hassles Scale (FCMAHS) measures CG burden in all aspects of medication management (MM)	*Administration*: Elevated scores in FCMAHS indicate higher burden with number of medications administered, *F*(1, 48) = 4.90, *p* = .03 ‐Caregivers administered an average of seven prescription medications and four over‐the‐counter medications (prescription medication: Mean = 7.37, SD = 3.66; over‐the‐counter medications: mean = 3.51, SD = 1.89) *Supply*: Over 90% of CGs collected medication [from pharmacists] (92.4%) and participated in medical appointments to maintain medication supply (98.1%) *Selection*: 83% CGs involved in decision‐making for dosage changes, medication initiation/cessation (86.8%)
Kurz, 2008, France, Germany, Spain, Brazil, North America^22^	Cross‐sectional	614	48	Survey identifies issues experienced by CGs in administration, monitoring and review	*Administration*: 1. Ensuring correct medication administration was the most important issue (83%–96%) 2. Improvements to MM: ‐Easier forms of medications (e.g., liquids) for easier administration (79%–99%) ‐More simplified dosage regimen to increase adherence (80%–96%) *Monitoring and review*: >90% CGs identified discussing treatment for the person with dementia with physicians as important ‐Most CGs preferred to avoid administering medicines that can cause adverse effects (79%–96%)
Levy and Steffen, 2016, USA^23^	Cross‐sectional	140	52.7	FCMAHS measures CG burden in all MM aspects; Medication Risk Questionnaire (MRQ8) identifies challenges in administration, supply, review	*Administration*: MRQ8: Most CGs administer five or more prescription medications (70%), 12 or more doses of medication daily (40%) and narrow therapeutic index medications (17%) ‐Caregivers administered on average seven prescription medications (mean = 6.82, SD = 3.9) and three over‐the‐counter medications (mean = 3.0, SD = 1.9) ‐CGs managed four or more changes to medications within the last year by physicians (44%) FCMAHS: More than 70% directly administered medications, organized medications (e.g., into pill planners) (87%) *Review*: MRQ8: Designed to identify older adults high risk for experiencing medication‐related problems, hence those who may benefit from medication evaluation *Selection*: FCMAHS: 77.1*%* involved in decision‐making for medication changes *Supply*: MRQ8: Majority of CGs manage prescriptions prescribed by multiple physicians (60%); over 25% obtain prescriptions at multiple pharmacies, have someone else collect medications (42%) FCMAHS: Most CGs ordered medications (80%) and collected medications (from pharmacists) (87%)
Lingler et al., 2016, USA^24^	Unblinded RCT	83	Intervention: 66.00 ± 12.8 Usual care: 67.80 ± 11.2	Medication Management Instrument for Deficiencies in the Elderly (MedMaIDE) appraises issues in administration, review, supply; Medication Deficiency Checklist (MDC) identifies CG deficiencies in administration and supply	*Administration*: Educational intervention on medication‐related problem solving had statistically significant improvements in CG administration techniques, ability to identify and count the correct number of tablets, indicated by decreased MedMaIDE scores post‐intervention (*F* = 6.91, *p* < .01) *Supply*: Statistically significant improvement in CGs' ability to identify prescription repeats, obtain new prescriptions and collect prescriptions (*p* < .01)
Noureldin and Plake, 2017, USA^5^	Cross‐sectional	1369	NR	Survey identified CG roles in MM	*Administration*: Approximately 50% of CGs assisted in ensuring correct times for administration to uphold adherence; very few CGs administered medications requiring injection (8.7%) *Monitoring and review*: Communicated with physicians regarding the PWD's pharmacotherapy (70%) *Supply*: Most CGs assisted in ordering medications (55.9%); only 9% managed prescriptions/medications
Piggott et al., 2017, USA^25^	Cross‐sectional	194	62	CG Confidence in Sign/Symptom Management (CCSM) Scale appraises CG confidence in selection, monitoring, review, administration	*Monitoring and review*: CGs with medical training were more confident in assessing and managing side effects, deciding when to contact physicians regarding medications for PWD
Schmidt, Steffen and Meuser, 2019, USA^26^	Cross‐sectional	152	50.2	MRQ8 identifies challenges in administration, supply, review; FCMAHS measures CG burden in all MM aspects	*Administration*: over 70% of CGs administered medication *Monitoring and review*: FCMAHS: Hassles in medication complexity (dosage regimens, forms, number of doses) (*r* = −0.07, *p* = .38) had a weak association with the risk of MRPs (*r* = .10, *p* = .24) *Supply*: Most CGs ordered (82.2%) and collected medication (87.5%) *Selection*: Most CGs involved in decisions about dosages (80.9%) and medication initiation/cessation (80.3%)
Travis et al., 2003, USA^27^	Mixed methods	158	61.1	FCMAHS appraises CG burden in all MM aspects; MCI identifies aspects of CG administration complexity	CGs who experienced increasing complexity in: *Administration*: dosages, forms, number of medications *Monitoring and review*: recognizing adverse effects *Supply*: obtaining new prescriptions and managing multiple prescriptions *Selection*: medication‐related decision‐making experienced greater burden in MM, as indicated by elevated FCMAHS and MCI scores
VanDyke and Steffen, 2017, USA^28^	Cross‐sectional	119	51.3	Survey appraises MM for CG in administration, selection, supply; MSB scale identifies CG issues in administration	*Administration*: Over 75% directly administered or reminded PWD to take their medications‐ Increased burden in administering and selecting medication was due to medication hoarding by care recipient, as indicated by higher MSB scores‐ Most CGs organized medications using dose administration devices (e.g., pill planners) (89.9%) *Selection*: Approximately 80% of CGs involved in decision‐making regarding medication changes *Supply*: CGs involved in ordering medication (86.6%) and collecting medication (92.4%)
Zimmerman et al., 2018, USA ^29^	Cross‐sectional	194	64	CCSM identifies CG confidence and responsiveness in selection, monitoring, review	*Monitoring and review*: Use of *AlzMed* improved CGs' confidence and ability to manage dementia and monitor medication side effects CG confidence improved over time after using *AlzMed* (mean CG confidence at baseline (3.8 ± 0.6 SD), 3 months (4.1 ± 0.7, *p* < .001) and 6 months (4.2 ± 0.5, *p* < .001) and was correlated with decrease in CG burden (mean CG burden at baseline (22.8 ± 7.2), 3 months (22.4 ± 8.6, *p* = .6) and 6 months (21.9 ± 7.8, *p* = .6)

Abbreviations: AlzMed, Alzheimer's medical advisor; CCSM, Caregiver Confidence in Sign/Symptom Management; CG, caregiver; FCMAHS, Family Caregiver Medication Administration Hassles Scale; MCI, Medication Complexity Index; MDC, Medication Deficiencies Checklist; MedMaIDE, Medication Management Instrument for Deficiencies in the Elderly; MM, medication management; MMT, medication management test; MRP, medication‐related problems; MRQ8, Medication Risk Questionnaire; MSB, medication‐saving behaviours; NR, Not reported in study; PWD, people with dementia; SD, standard deviation.

### 3.2 Medication administration

Medication administration was an aspect of medication management that was reported in 9 out of 10 studies^5^, ^21^, ^22^, ^23^, ^24^, ^25^, ^26^, ^27^, ^28^ (Table 1). The Medication Risk Questionnaire evaluated administration challenges with regard to the number of medications that caregivers manage (five or more prescription medications [70%]), number of doses administered daily (12 or more doses daily [40%]) and whether caregivers administer medications with a narrow therapeutic index (e.g., phenytoin, lithium) (17%).^23^ The Hassles scale evaluated caregivers managing administration schedules, preparation of medicines and challenges in administering medications in a person living with dementia.^27^ Forgetting to administer medication, dropping or losing medication or administering the wrong dose from the dose administration aid were the most common caregiver administration errors identified from the Medication Deficiency Checklist.^24^ Two studies distinguished between caregivers providing direct administration to people living with dementia, or assisting in administration through reminding the person with dementia to take their medications.^5^, ^28^ The survey used by Noureldin and Plake^5^ identified whether caregivers administered medications via different routes.

Caregiver involvement in medication administration showed a wide range, from 44% to 94.7%, across nine studies.^5^, ^21^, ^22^, ^23^, ^24^, ^25^, ^26^, ^27^, ^28^ Two studies reported on administration in relation to the number of medications administered.^21^, ^23^ Across the two studies, caregivers reported being responsible for administering an average of seven prescription medications and four over‐the‐counter medications.^21^, ^23^ George and Steffen^21^ reported that the number of medications administered was significantly correlated with the degree of caregiver burden in medication management, indicated by elevated Hassles scale scores (*p* = .03). Caregivers experienced a greater burden in medication management when complexity in administration increased, due to different medication dosages, forms and number of medications.^27^ Levy and Steffen^23^ reported that nearly half of caregivers managed four or more changes to the person with dementia medications or instructions for administration, within a 1‐year time frame (44%). Two studies identified that most caregivers prepared medicine for administration by organizing medication with dose administration devices (87%–89.9%).^23^, ^28^ A multicountry survey reported that ensuring correct administration was considered most important in medication management (83%–96%) by caregivers and suggested easier forms of medications (79%–99%) and simplified dosage regimens (80%–96%) to improve administration.^22^

### 3.3 Medication monitoring and review

Caregiver involvement in medication monitoring and review was captured in 7 out of 10 studies using the Caregiver Confidence in Sign/Symptom Management scale, the Medication Management Instrument for Deficiencies in the Elderly, the Hassles scale and the Medication Risk Questionnaire, whilst some studies used surveys (Table 1).^5^, ^22^, ^23^, ^24^, ^25^, ^26^, ^27^, ^29^ The Hassles scale contained a subscale that evaluated caregiver burden in medication review.^27^ These items included being comfortable in speaking with physicians about medications for the person with dementia, knowing if the medication is having the desired effect and adverse effects to monitor for.^27^ The Caregiver Confidence in Sign/Symptom Management scale used a subscale to evaluate the confidence and responsiveness of the caregiver, with an item relating to caregivers' decision‐making for when to contact medical providers regarding general medication management for the person with dementia.^25^, ^29^ The Medication Risk Questionnaire can identify individuals living with dementia who are high risk for developing medication‐related problems,^23^ such as individuals exposed to polypharmacy (more than five prescription medications) and those taking high‐risk medications for medication‐related problems.^23^

Kurz et al.^22^ reported that more than 90% of caregivers believed that it was very important within their role to discuss the condition and treatment of the person with dementia with the physician. Caregivers were risk‐averse to administering medications that can produce adverse effects (79%–96%), believing that better control of dementia symptoms (94%–100%) and having less bothersome side effects (84%–100%) were also important issues in medication management.^22^ Two studies observed that caregivers with greater medical training were more confident in evaluating and managing side effects for people with dementia in community settings, and knowing when and how to review medications with medical providers.^25^, ^29^ Lingler et al.^24^ found that interventions that educated caregivers on medication side effect monitoring and administration routes improved caregiver knowledge (*p* < .01).

### 3.4 Medication selection

A total of 6 out of 10 studies identified selecting medications as an aspect of caregiver medication management, using the Medication Risk Questionnaire and Hassles scale tools, with one study also having used a survey^21^, ^23^, ^26^, ^27^, ^28^, ^29^ (Table 1). The Hassles scale and a survey specifically evaluated all aspects of medication selection ‐ initiating and ceasing medicines, or changing doses.^26^ The Caregiver Confidence in Sign/Symptom Management scale was unique by evaluating caregivers' decision‐making and confidence to select medicines for the person with dementia, depending on the development of cognitive and medication‐related symptoms.^25^, ^29^

Most caregivers were involved in decision‐making regarding medication changes for the person with dementia (77.1%–86.8%).^21^, ^23^, ^28^ Schmidt et al.^26^ reported that almost an equal proportion of 152 caregivers were involved in decisions about dosages (80.9%) as they were involved in decisions to initiate/cease medications (80.3%). This finding was similarly reported by George and Steffen, who found that most caregivers in the study were involved in decisions regarding dosage changes (83.0%) and in initiating/ceasing medications for the person living with dementia (86.8%).^21^ Travis et al. reported a correlation between increased complexity in medication selection (medication‐related decision‐making) and increased caregiver burden in medication management, which was indicated by elevated Hassles and Medication Complexity Index scores (Medication Complexity Index, *r* = .19, *p* = .05; Hassles Caregiver Strain Index, *r* = .44, *p* = .01).^27^ Caregiver confidence improved after an educational intervention on medication‐related problems, evaluating vital observations and managing medications for pain (mean caregiver confidence at baseline (3.8 ± 0.6 SD), 3 months (4.1 ± 0.7, *p* < .001) and 6 months (4.2 ± 0.5, *p* < .001) (Table 1).^29^ Increased caregiver confidence was negatively correlated with a small decrease in caregiver burden over time, but this was not statistically significant (mean caregiver burden at baseline (22.8 ± 7.2), 3 months (22.4 ± 8.6, *p* = .6) and 6 months (21.9 ± 7.8, *p* = .6) (Table 1).^29^

### 3.5 Medication supply

Supply was a subcomponent of medication management in seven studies^5^, ^21^, ^23^, ^24^, ^26^, ^27^, ^28^ (Table 1). The Medication Risk Questionnaire identified caregiver management of medication supply with an item relating to whether caregivers manage prescriptions prescribed by multiple physicians (60%).^23^ The Hassles scale and Medication Complexity Index evaluated the implications of complexity in supply due to obtaining new prescriptions and managing multiple prescriptions on caregiver burden in medication management.^27^ Interestingly, the Medication Risk Questionnaire was the only tool to explore medication supply with an item that asked if prescriptions are procured from multiple pharmacies (26%) and whether an individual besides caregivers is responsible for medication collection and delivery to home (42%).^23^ It is plausible that having a third party collect medications from multiple pharmacies can be a risk factor for medication‐related problems, due to miscommunication or lack of continuity of care from pharmacist to caregiver/person with dementia.

Five studies reported that the majority of caregivers were involved in ordering medications (55.9%–86.0%).^5^, ^23^, ^24^, ^26^, ^28^ Four of these studies, including Noureldin and Plake, found that most caregivers were responsible for collecting medications from pharmacies (87%–92.4%).^21^, ^23^, ^24^, ^26^, ^28^ Three studies identified that caregiver management of medication supply involved managing prescription supplies, often from multiple physicians (60%).^5^, ^23^, ^24^ Increasing complexity in medication supply increased caregiver burden in medication management.^27^ Lingler et al.^24^ found that education on medication management resulted in a statistically significant improvement in caregivers' ability to identify prescription repeats, collect and obtain new prescriptions (*p* < .01).

### 3.6 Reliability and validity of tools and surveys

Table 2 describes the reliability and validity of the tools. Most tools had reliability and validity information, except for the Medication Complexity Index.^27^ Surveys did not have reliability or validity information. All tools reported good reliability, except for the Medication Deficiency Checklist, which had a low internal consistency (Cronbach's alpha = .38).^24^ All tools were validated, except for the Medication Deficiency Checklist. Most tools assess validity by reporting on subscale intercorrelation (using Pearson's correlation) and overall tool score correlations.

**Table 2:** Reliability and validity information of the tools included in the review

Tool^a^	Reliability data	Validity data
Caregiver Confidence in Sign/Symptom Management	*Internal consistency*: Cronbach's *α* = 0.92 *Test–retest reliability: r* = .92 *Intraclass correlation coefficient: r* = .91	*Convergent and concurrent validity*: Positive correlation between overall CCSM scale scores and caregivers with medical training (*r* = .26, *p* < .001)
Family Caregiver Medication Administration Hassles Scale	*Internal consistency*: Cronbach's *α* = 0.95 *Test–retest reliability: r* = .84	*Discriminant validity*: Intercorrelations between subscales and total Hassles scale: –Information seeking/sharing (*r* = .86) –Scheduling logistics (*r* = .83) –Safety issues (*r* = .80) –Polypharmacy (*r* = .77) *Construct validity*: Modest measure of concurrent validity for the Hassles scale correlation with the MCI score and the Caregiver Strain Index (total Hassles scale score): –MCI (*r* = .19, *p* = .05) –CSI (*r* = .44, *p* = .01)
Medication Deficiency Checklist	*Internal consistency*: Cronbach's *α* = .38 *Test–retest reliability: r* = .66, *p* < .001	*Concurrent validity*: Negative correlation between medication adherence and MDC scores (*r* = −.30, *p* < .001)
Medication Management Instrument for Deficiencies in the Elderly	*Internal consistency*: Cronbach's *α* = .71 *Test–retest reliability: r* = .93 *Interrater reliability*: Intraclass correlation = .74	*Concurrent validity*: Modest correlation between MedMaIDE scores and pill count compliance (*r* = −0.52) *Predictive validity*: Positive predictive value for MedMaIDE scores (0 vs. 1+ = 0.65; 0–1 vs. 2+ = 0.83), thus higher probability that MedMaIDE could identify deficiencies in medication management compared to pill count measure
Medication Risk Questionnaire	*Internal consistency*: Cronbach's *α* = .69 *Test–retest reliability: κ* > 0.6	*Criterion validity*: Significant positive correlation between MRQ8 scores and health‐related concerns of older adults (e.g., falls in the past year, injuries) –Multivariate analysis of the high‐risk group comparison (*p* < .01) supported clinical cutoff for MRQ8 score = 4, indicative of moderate–serious risk of associated negative health concerns
Medication‐Saving Behaviours	*Internal consistency*: Cronbach's *α* = .85	*Content validity*: Correlations between scale items and loading factors reported *Convergent and discriminant validity*: Lack of relationship with the overall number of prescription and nonprescription (over‐the‐counter) medications currently taken

Abbreviations: CCSM, Caregiver Confidence in Sign/Symptom Management; FCMAHS, Family Caregiver Medication Administration Hassles Scale; MDC, Medication Deficiencies Checklist; MedMaIDE, Medication Management Instrument for Deficiencies in the Elderly; MRQ8, Medication Risk Questionnaire; MSB, medication‐saving behaviours.

^a^ All tools, except the Medication Complexity Index, provided reliability and validity data. All surveys did not report reliability and validity data. For this reason, surveys have not been included in this table.

## 4 DISCUSSION

To our knowledge, this is the first systematic review to identify approaches used to evaluate medication management for caregivers of people living with dementia. While most tools and surveys evaluated multiple aspects of medication management, they did not comprehensively assess the full range of complexities for caregivers managing medications for people living with dementia.

Our findings suggest that up to 95% of caregivers reported that they were involved in medication administration.^5^, ^21^, ^22^, ^23^, ^24^, ^25^, ^26^, ^27^, ^28^ Administration of medications was highlighted as a challenging aspect for caregivers due to concerns in managing multiple medications whilst avoiding medication errors that might compromise the safety of the person with dementia.^5^, ^30^ However, very few tools and surveys evaluated factors that contribute to complexities in medication management. The Medication Risk Questionnaire was one of the more unique tools as it evaluated administration challenges due to polypharmacy and more complex dosing regimens.^23^ This was demonstrated by 70% of caregivers administering medications to people living with dementia taking five or more prescription medications, and 40% of caregivers administering 12 or more doses daily.^23^ Managing polypharmacy is a common issue contributing to administration burden in caregivers.^31^ Qualitative studies highlight that other factors contribute to complexities in medication management, such as limited medication information provided at transitions of care, limited caregiver involvement in shared decision‐making and feeling unsupported.^15^, ^32^, ^33^, ^34^ Therefore, a tool that evaluates these factors is needed to identify areas for improvement to reduce medication‐related adverse effects in people living with dementia.^35^

The Hassles scale evaluated errors in administration with items on the degree of caregiver burden in relation to caregivers admitting to physicians when they have made medication errors and knowing how to safely administer medication.^27^ The Medication Deficiency Checklist identified that the most common errors in medication administration by caregivers are forgetting to administer medication, losing/dropping medication or administering the wrong dose.^24^ However, the included studies did not explore the reasons for administration errors. Vision and dexterity issues and decline in cognitive function may contribute to medication errors.^19^, ^36^, ^37^, ^38^ Other factors include polypharmacy, lack of medical training and feeling overwhelmed from medication burden.^32^, ^34^ Horne et al.^1^ reported that caregivers lacked awareness of medication aids that could improve adherence, and interventions that raised awareness were seen as beneficial in reducing medication management burden. Therefore, a tool that assesses caregiver knowledge and utilization of medication aids is needed to identify solutions to improve medication management. This will inform understanding of the guidance that caregivers require from healthcare professionals, such as providing advice on using reminders for medication administration times or the use of medication aids to overcome dexterity issues for caregivers.

A recent systematic review found that multidisciplinary interventions that extend beyond hospital discharge to guide caregivers in medication management for individuals with dementia are important.^34^ However, none of the included studies have explored using different primary healthcare professionals as sources of medication information. Caregivers often seek medication information from physicians, but limited appointment timeframes^32^ or lack of involvement in decisions and discussions have been cited as barriers that contribute to increased caregiver burden.^19^, ^36^, ^39^ Terayama et al.^40^ reported that a multidisciplinary approach by doctors, nurses and social workers in providing education classes to caregivers of people with dementia, such as on management of dementia symptoms and medication adherence, resulted in significant improvement in caregiver burden and depression compared to controls. Similarly, a multidisciplinary intervention that addressed caregiver–patient needs at transitions of care, such as medication management, reduced rehospitalization rates for care recipients at 1 and 3 months post‐discharge (*p* = .04) compared to controls, whilst also decreasing the mean hospital costs for the intervention group ($2546 control vs. $2058 intervention patients, *p* = .05).^41^ In addition, pharmacists have the appropriate medication knowledge and are the most accessible healthcare professionals, ideally placed in community settings, to deliver medication information to caregivers.^42^ This is particularly relevant during transitions of care when caregivers can experience an increased need to consult with healthcare professionals regarding medications for their care recipient.^41^ Over 90% of caregivers in this review were from a community setting, and most visit pharmacies as they collect medications for people living with dementia. Guidance from pharmacists can reduce the burden in medication management for caregivers and improve the care provided for people living with dementia.^35^ Therefore, a tool is needed to evaluate caregiver access to multidisciplinary healthcare to receive advice on medication management.

### 4.1 Strengths and limitations

The major strength in this review was the rigorous and extensive database search completed across five databases, with detailed variations of search terms, particularly for ‘medication management’. This enabled us to capture as many published studies as possible containing aspects of caregiver medication management more holistically, therefore providing an accurate systematic review reflecting the available literature.

Another strength of this review was the reliability of the tools. Most tools had good reliability, and all tools except one provided validity information that, for the purpose of this review, enabled a greater understanding of whether the tool was able to measure aspects of medication management for caregivers. An area of improvement would be to adapt a validated scale, such as the Hassles scale, and add items pertaining to medication management for caregivers of people living with dementia at transitions of care. There is limited understanding of caregivers' experience of medication management guidance for people living with dementia during care transitions.^19^ Thus, this would improve knowledge of caregivers' medication management for people living with dementia.

However, this review had several limitations. The major limitation was that all studies are conducted in the United States of America; consequently, there is a lack of data from different countries to determine if evaluation of caregiver medication management differs or is similarly reported. Although the Hassles scale was the only tool that evaluated all aspects of medication management, it was limited in that the aspects were only evaluated in terms of caregiver burden.^21^, ^23^, ^26^, ^27^ Consequently, it does not provide a holistic view of caregiver involvement in different aspects of medication management. Therefore, further work is needed on developing a tool with more items to holistically evaluate the complexities of caregiver medication management. Another limitation was the lack of exploration of causes for the complexities in medication management within the included studies. Therefore, an area of further research could involve including qualitative studies on medication management for caregivers of people living with dementia to explore the complexities contributing to medication administration issues.

Another limitation was that there can be a risk of bias due to the inclusion criteria for the included studies. Although the review had a rigorous database search strategy, we may have missed studies due to the inclusion of only those published in English and that were quantitative studies. Trials' registries and dissertations were not searched, which can further increase the risk of missed studies for the review. Most studies also had small sample sizes, further biasing the results for the review. Another limitation was that some studies used the tools and surveys as a subcomponent of an analysis into an intervention, whereas for some, it was the study focus. Consequently, these studies did not report all findings from the tool, instead focusing on certain aspects of the tool and its respective results. Two studies—Levy and Steffen^23^ and Schmidt et al.^26^—both used the Medication Risk Questionnaire; however, the latter study did not present the questionnaire results, as it was a subcomponent tool to evaluate the validity and reliability of another screening tool, which was the study focus.^26^ This created challenges in comparing and supporting results on aspects of medication management between different studies using the same tools. It also impaired the ability to ascertain the data needed to appraise caregiver involvement in medication management.

## 5 CONCLUSIONS

This systematic review identified and summarized tools to evaluate medication management for caregivers of people living with dementia, and appraised caregiver involvement in aspects of medication management. The findings suggest that there is limited knowledge on the medication management provided by caregivers of people living with dementia across countries, with most studies identified in this review being conducted in the United States of America. Of the available tools identified in this review, the Hassles scale appears to be best suited to evaluate the medication management provided by caregivers of people living with dementia. Administration emerged as the most appraised aspect of medication management in the literature, with challenges in administration contributing to caregiver burden. It is evident that a tool is needed to holistically evaluate medication management and its complexities for caregivers of people living with dementia, which can be used across different settings and at transitions of care. This will aid in the development of strategies to address these complexities in medication management, which will help to ensure that people living with dementia receive safe and effective care. In so doing, it will help uphold optimal health outcomes and quality of life, standardize medication management across transitions of care and decrease caregiver burden in medication management.

## CONFLICT OF INTERESTS

The authors declare that there are no conflict of interests.

## AUTHOR CONTRIBUTIONS

*Study concept and design*: Dr. Danijela Gnjidic and Dr. Mouna Sawan. *Acquisition of data*: Melissa Gench and Aili Langford. *Analysis and interpretation of data*: Melissa Gench, Dr. Mouna Sawan and Dr. Danijela Gnjidic. *Preparation of the manuscript*: Melissa Gench, Dr. Mouna Sawan, Dr. Danijela Gnjidic and Aili Langford.

## Data Availability

The data that support the findings of this study are available from the corresponding author upon reasonable request.
